# Robot‐assisted laparoscopic augmentation ileocystoplasty and Mitrofanoff appendicovesicostomy in children: Step‐by‐step and modifications to UChicago technique

**DOI:** 10.1002/bco2.7

**Published:** 2020-03-20

**Authors:** Brittany Adamic, Lakshmi Kirkire, Ciro Andolfi, Craig Labbate, Joshua Aizen, Mohan Gundeti

**Affiliations:** ^1^ Pediatric Urology Section of Urology Department of Surgery Comer Children's Hospital The University of Chicago Pritzker School of Medicine Chicago IL USA; ^2^ The University of Chicago Pritzker School of Medicine Chicago IL USA

**Keywords:** augmentation ileocystoplasty, neurogenic bladder, pediatric, robotic

## Abstract

**Objective:**

To describe the step‐by‐step techniques and modifications for robot‐assisted augmentation ileocystoplasty and Mitrofanoff appendicovesicostomy in a pediatric population with updated institutional results.

**Introduction:**

Robot‐assisted laparoscopic augmentation ileocystoplasty with Mitrofanoff appendicovesicostomy (RALIMA) protects the upper urinary tract and reestablishes continence in patients with refractory neurogenic bladder. Robotic assistance could provide the benefits of minimally invasive surgery without the challenges of pure laparoscopy. Here, we focus on the outcomes of RALIMA with salient tips and modifications of the technique.

**Methods:**

We performed a retrospective review of our robotic database and identified 24 patients who underwent attempted robot‐assisted laparoscopic augmentation ileocystoplasty (RALI) between 2008 and 2017 by a single surgeon at an academic center. Outcomes of interest included operative time, hospitalization time, postoperative complications, and change in bladder capacity. RALI and all concomitant procedures were performed using the da Vinci® surgical system (Intuitive Surgical, Sunnyvale, CA, USA).

**Results:**

Of 24 patients, 20 successfully underwent RALI. Eighty percent underwent concomitant appendicovesicostomy (APV), 40% underwent antegrade continence enema channel formation (ACE), and 30% underwent a bladder neck procedure. Mean operative time was 573 minutes and the most recent RALIMA was 360 minutes. The average return to regular diet was 3.9 days and length of stay was 6.9 days. Mean change in bladder capacity was 244% postoperatively. Thirty‐day complications were noted in 35% of patients; one Clavian grade I (5%) complication, five grade II (25%) complications, and one grade IIIb (5%) complication. With a median follow‐up of 83.1 months we note a 25% incidence of bladder stones, 15% upper tract stones, 5% incidence of bladder rupture, and 5% small bowel obstruction. No patients required re‐augmentation in the follow‐up period.

**Conclusions:**

RALI has similar functional outcomes and complications when compared with the open augmentation ileocystoplasty literature. RALI is desirable due to favorable pain control with decreased length of stay. Long‐term outcomes after RALI are similar to the open approach. As the operative time is currently the largest point of criticism with the robotic approach, we discuss modifications to decrease the operative time.

## 1 INTRODUCTION

Augmentation ileocystoplasty is necessary to protect the upper tracts in patients who fail medical management of their neurogenic bladders. Indications include severe hydronephrosis and/or concerning urodynamic parameters such as detrusor leak point pressure ≥40 cm H_2_O in patients who have failed medical management. Augmentation ileocystoplasty leads to decreased voiding pressures as well as increased bladder capacity and continence. The majority of patients undergoing this procedure have neurogenic bladder or valve bladder due to posterior urethral valves.^1^ Additional abnormalities that account for the condition include the spectrum of spina bifida.^1^, ^2^

In the majority of cases, augmentation ileocystoplasty is coupled with the creation of a continent catheterizable channel (CCC) which allows for patients to achieve social continence and to avoid urethral catheterization. Urethral catheterization can ultimately cause discomfort in a sensitive urethra as well as complications such as trauma, strictures, and eventually patient noncompliance.^1^ Most often, the appendix is used for the CCC (Mitrofanoff appendicovescostomy) but another common technique is using a transversely tubularized segment of bowel as a channel (Yang‐Monti technique).

Augmentation ileocystoplasty is traditionally performed as an open procedure. While a laparoscopic approach has been described, the steep learning curve has prevented its incorporation into widespread practice.^3^ Since the later introduction of the robotic approach, it has been increasing in prevalence although the technical demands of the operation limit universal adoption.

We present an updated technical demonstration video of RALIMA on a pediatric patient, with step‐by‐step methodology of the operation with modifications since its inception in 2008 (UChicago technique) as well as updated long‐term outcomes at our institution.^4^

## 2 SURGICAL TECHNIQUE

*Indicates modified technique.

### 2.1 Preoperative preparation

We do not use a bowel preparation preoperatively.^5^, ^6^, ^7^ Patients are encouraged to continue a regular diet until midnight the night before surgery. Patients are admitted to the hospital postoperatively on the day of surgery. A preoperative antibiotic protocol of weight‐based cefazolin, metronidazole, and gentamicin is administered within the 30 minutes before skin incision and are continued for 24‐48 hours. Patients with ventriculoperitoneal shunts also receive one dose of prophylactic vancomycin preoperatively. A neurosurgery consult is obtained prior to surgery for these patients.

### 2.2 Patient positioning, port placement, and robot docking

The patient is positioned in a supine semilithotomy with 10 degrees Trendelenburg. A Foley catheter is placed sterilely on the field. A nasogastric tube is inserted for the duration of the surgical procedure. A 12‐mm camera port is placed in a supraumbilical position using Hasson's technique*. We have previously described umbilical camera port placement; however, we find the supraumbilical port placement allows for easy identification and dissection of the appendix and bowel. If utilizing the DaVinci X or Xi® robot, an 8‐mm camera port is placed in a similar position. After establishing pneumoperitoneum, the 8‐mm robotic working arm ports are placed laterally at the level of the umbilicus in the midclavicular line. A 5‐mm assistant port is placed in the left upper quadrant, inferior to the costal margin and in the midclavicular line (this can be substituted for a large assistant port if staples are utilized for bowel anastomosis). A fourth robotic working arm port can be placed at the site of stoma creation in the right iliac fossa for patients who are greater than 12 years of age or 5 feet tall due to space restriction.

### 2.3 Diagnostic peritoneoscopy

We recommend beginning the case with diagnostic peritoneoscopy and lysis of adhesions if necessary. This step facilitates the ease of appendix isolation, especially the suprahepatic locations in patients with VP shunts. If present, a VP shunt can be placed into an endocatch bag. The appendix is identified, ensuring adequate length and vascularity to allow for successful appendicovesicostomy. The evaluation of the appendix and intra‐abdominal anatomy allows for open conversion if required prior to docking the robot. If appropriate expertise is available, a Monti channel can be created robotically.

### 2.4 Appendiceal isolation and harvest

A traction suture can be placed at the tip of the appendix to aid in dissection and manipulation. A 4‐0 Vicryl suture (polyglactin) is placed as a stay suture and a mesenteric window with adequate blood supply is developed. The appendix is then excised from the cecum. If a short appendix is noted, a cecal flap can be created to ensure adequate length and to avoid stomal stenosis.* In those who require antegrade continence enema channel creation, the length of the appendix will determine the need for a split technique vs a cecal flap. The defect in the cecum is closed in two layers.

### 2.5 Ileal loop isolation and anastomosis

A 20‐cm ileal segment is isolated 20 cm proximal to the ileocecal junction for the cystoplasty patch. Percutaneous stay stitches placed in the proximal and distal ends of the bowel are performed with Keith needles. This maneuver provides traction of the bowel and allows for easier isolation and anastomosis. A premeasured umbilical tape is used to ensure accurate measurement of the bowel segments. After ensuring mesenteric length and that the ileal segment will reach the bladder, the ileal loop is transected. We demonstrate the division of the mesentery with Harmonic scalpel®* and bipolar forceps to reduce bleeding and facilitate the dissection.

Bowel continuity is re‐established by hand sewn single‐layer seromuscular anastomosis using 5‐0 PDS in children, or 4‐0 PDS in adults. We start the anastomosis on the antimesenteric border using a running stitch toward the mesentery on the posterior wall. On the anterior wall of the bowel, a separate stitch is run from the mesenteric border toward the antimesenteric border. The mesenteric defect is closed to prevent the possibility of closed loop bowel obstruction.

### 2.6 Detrusorotomy and extravesical appendicovesicostomy

In the case of a short appendix (as such in the video), an oblique extravesical appendicovesicostomy with stoma formation in the right iliac fossa can be created. Otherwise, we suggest the intravesical approach with posterior wall implantation to reduce the operative time.* The detrusorotomy can be made in the coronal plane to reduce bleeding.* When performing the intravesical approach, the appendix is brought to the posterior wall and oriented according to planned stoma site creation (umbilical location proceed with midline anastomosis, while right iliac fossa requires an oblique anastomosis). The bladder is distended with sterile saline and a detrusorotomy is made along the posterior bladder wall in the coronal plane. The previously placed stay suture at the tip of the appendix allows for easy manipulation while minimizing direct handling of the appendix. The appendix is spatulated and anastomosed to the bladder mucosa with interrupted 5′0 PDS II® Medline Industries (polydioxanone) stitches. An 8 French feeding tube is placed within the appendix. After the anastomosis is performed, the detrusor muscle is closed over the appendix with 3‐0 or 4‐0 Vicryl in continuous fashion without tightening. We do not fenestrate the mesentery or tack the appendix to the bladder wall.

### 2.7 Ileal detubularization

The previously isolated ileal segment is now detubularized along the antimesenteric border with a harmonic scalpel*. This allows for a reduction in operative time by reducing the bleeding. Stay sutures are placed at the proximal and distal ends of the ileal patch to prevent torsion of the mesentery.

### 2.8 Cystotomy and patch ileoystoplasty

The cystotomy is performed in the coronal plane. A thick‐walled bladder is often encountered, and we find the harmonic scalpel aids in hemostasis and decreases operative time compared to our previous use of monopolar scissors.* We then turn attention to the augmentation with ileal patch. The detubularized bowel is sutured to the apices of the cystotomy. Utilization of the 4th arm can aid in retraction and exposure. We now use a barbed quill suture to perform the posterior bowel bladder anastomosis in a continuous fashion.* We utilize either a 2‐0 Quill^TM^ suture (Surgical specialties corporation), Vicryl, or polydioxanone (PDS). In our experience, placement of only one suprapubic catheter often leads to dislodgement and clogging; therefore, two suprapubic catheters are placed percutaneously to provide maximal drainage.* The anterior bladder bowel anastomosis is then performed, working from the apices toward the midline. The augmented bladder is filled with sterile water to identify leakage.

### 2.9 Maturation of appendix stoma to right iliac fossa

The appendix can be brought to the predetermined stoma site with the assistance of a stay suture. A skin flap (V, VQ, VQZ technique) is created and anastomosed to the Mitrofanoff appendicovesicostomy (MAPV) using 5‐0 PDS II® Medline Industries (polydioxanone) suture. The fascia of the remaining port sites is closed with 2‐0 Vicryl suture under direct vision and the skin is closed with 5‐0 Monocryl® Ethicon (poliglecaprone) or equivalent suture.

### 2.10 Postoperative management

All catheters (two suprapubic catheters and one urethral catheter) are left to freely drain for 4 weeks. The appendicovesicostomy catheter is capped and secured to the abdomen. At 4 weeks postoperation, the MAPV catheter is removed and the patient/family is taught clean intermittent catheterization (CIC). The suprapubic catheters are removed after the patient/family has shown competence in performing CIC.

## 3 METHODS

After obtaining approval from our institutional IRB, we identified patients who underwent robot‐assisted laparoscopic ileocystoplasty (RALI) at our institution between 2008 and 2017 by a single surgeon. Four patients were excluded due to conversion to open surgery. Preoperative videourodynamics and renal ultrasounds were performed on all patients, with adjunctive imaging, such as VCUG and DMSA scans as indicated postoperatively; serial renal ultrasounds and metabolic evaluation were also performed on all patients. Urodynamics and any adjunctive tests were used as needed. Demographic information, preoperative UDS findings, operative details, and postoperative outcomes including complications were recorded and are described.

## 4 RESULTS

A total of 24 patients were scheduled to undergo RALI; however, four were converted to open surgery and therefore excluded from the final analysis. Reasons for open conversion include two patients with kyphosis limiting intra‐abdominal space on insufflation and two patients with dense adhesions and unfavorable appendiceal anatomy. Table 1 displays patient characteristics. The median age was 11.7 years old and 60% of patients were male. The median weight in kilograms was 44.3 and median BMI was 19.9. Eight patients (40%) had VP shunts. Only two patients (10%) had a history of posterior urethral valves (PUV). Table 2 depicts perioperative details. Mean operative time was 573 minutes; however, this is difficult to quantitate due to 11 patients undergoing concomitant procedures. Operative time includes skin incision and ends after skin closure and includes docking of the robot. Our most recent patient underwent RALIMA with an operative time of 360 minutes. Mean time to regular diet was 3.9 days and mean length of stay was 6.9 days. Mean change in bladder capacity was increased by 244% postoperatively which is displayed in Table 3.

**Table 1:** Patient Data and Complications

Age at surgery (years)	Gender	Weight (kg)	Pathology	OR time (mins)	Additional procedure	Preoperative bladder capacity (mL)	Postoperative bladder capacity (mL)	Regular diet (days)	Length of stay (days)	0‐30‐day complications	30‐90‐day complications	>90‐day complications	Follow up (mos)	Secondary surgery (mos)
14.8	Male	44.2	PUV	379	APV	618	600	6	7	UTI	UTI, difficulty catheterizing	UTI	35.7	Mitrofanoffscopy (1.2)
8.8	Female	28.6	Sacral agenesis	480	ACE, APV	90	400	4	6	None	None	UTI, bladder stone	111.2	ACE deflux (6), Mitrofanoffscopy (12), ACE takedown (73)
14.2	Male	44.5	Spina bifida, sacral agenesis	573	ACE, APV	171	600	14	16	pSBO requiring TPN and NGT, UTI	Bladder perforation	Bladder stone	41.9	Endoscopic cystolitholapaxy (8), Mitrofanoffscopy (21)
14.9	Male	54.4	Myelomeningocele	512	ACE, Urethral Sling	229	500	3	8	None	None	Urethral sling erosion	36.3	Removal eroded sling and urethroplasty (10), SPT and bladder neck deflux (12), pyeloplasty (31)
15.6	Male	56.7	Myelomeningocele	506	ACE, APV	180	500	4	7	UTI	None	UTI	17	None
9.3	Female	24.6	Meningomyelocele	509	ACE, APV	105	300	4	6	None	UTI, difficulty catheterizing	UTI, difficulty catheterizing	28.3	Mitrofanoffscopy (2), Open Monti channel creation (54)
13.8	Female	72.8	Myelomeningocele, open bladder neck	659	APV, BNC	50	275	3	6	None	None	UTI	81.4	PCNL (82)
25.6	Male	56.7	Sacral agenesis, open bladder neck, high‐level imperforate anus	708	Urethral sling	46	350	2	4	None	None	None	28.6	Umbilical Sinus exploration (24)
11.8	Male	36.3	Tethered cord, poor compliance, open bladder neck	532	APV, BNC, circumcision	87	400	5	7	Bladder neck dehiscence	None	Bladder neck dehiscence, UTI	95.6	Mitrofanoffscopy and SPT (<1), Bladder neck closure (3), Stomal polyp excision‐skin level (59)
7.6	Male	34.5	Myelomeningocele, open bladder neck	923	APV, ACE, BNC	140	250	6	8	None	None	UTI, bladder neck dehiscence	96.7	Bladder neck closure (36), Endoscopic cystolitholapaxy (62)
11.7	Female	34.3	Myelomeningocele, open bladder neck	654	APV, ACE, BNC	60	500	2	4	None	None	UTI, bladder neck dehiscence, bladder stone	86.9	Bladder neck closure, re‐do ACE (21) Attempted staged endoscopic cystolitholapaxy (32), Open cystolitholapaxy (33)
11.8	Male	65.7	Posterior Urethral Valves	467	APV	450	550	4	5	None	None	UTI	84.9	Kidney transplant (22), Mitrofanoffscopy (47)
7.7	Male	25.2	Myelomeningocele	627	ACE	90	300	3	7	None	None	Urethral leakage of urine (large bladder neck requiring multiple procedures), bladder stone	117.6	Bladder neck deflux (5,6), Monti channel, Bladder neck reconstruction and Bilateral ureteral reimplantation (12), Bladder neck closure (20), Endosopic cystolitholapaxy (10,70,102) Open cystolitholapaxy (48, 55, 97)
7.4	Male	23.4	Tethered cord	711	APV	110	700	4	7	Ileus	None	UTI, keloid formation, parastomal hernia	120.1	Mitrofanoffscopy (1.1, 13), Stoma revision (3.4), Parastomal Hernia repair (33)
11.8	Female	34	Sacral agenesis	610	APV	170	300	1	6	None	difficulty catheterizing	UTI, APV leakage	126.2	Mitrofanoffscopy (1.6), Deflux into APV (12)
10.4	Female	40	Myelomeningocele	609	APV	250	450	1	8	SSI, Paresthesia	None	UTI, stomal stenosis, keloid formation, false passage	129.4	Stoma revision (5,18), Mitrofanoffscopy (17, 17, 37, 42)
8.1	Male	37.8	Myelomeningocele	532	None	100	400	2	6 (15)	DVT	None	None	1.5	None
10.8	Female	51	Myelomeningocele	646	APV	220	500	4	6	None	None	Stenosis of APV	124.1	Stoma Revision (>3)
16.7	Female	64.7	Lipomyelomeningocele	465	APV	120	375	4	8	None	None	Ureteral stone, bladder perforation	6	Ureteroscopy (6)
17.9	Male	52.6	Microcephaly, Developmental delay, Chromosomal abnormality	360	APV	###	500	5	7	None	difficulty catheterizing	SBO	15.3	Mitrofanoffscopy (1.4), Stoma revision (2.7), Exploratory laparotomy for bowel obstruction (13)

**Table 2:** Perioperative and hospital data

Perioperative and hospital data	
*Concomitant procedures (%)*	
Appendicovesicostomy	16 (80)
Antegrade continence enema	8 (40)
Bladder neck procedure	6 (30)
OR time, minutes (95% CI)	573 (360‐633)
EBL, mL (95% CI)	103 (64‐142)
Regular diet (95% CI)	3.9 (2.6‐5.2)
Length of stay, days (95% CI)	6.9 (5.8‐8)

**Table 3:** Change in Bladder Capacity

Change in bladder capacity	
Preoperative capacity, mL (95% CI)	214 (106‐322)
Percent of expected (95% CI)	51 (30‐72)
Postoperative capacity, mL (95% CI)	437 (380‐494)
Percent change capacity (95% CI)	244 (143‐345)

Complications are described in Table 4. Early complications (30 days postoperatively) were identified in seven (35%) patients. There was one Clavian grade I (5%) complication, five grade II (25%) complications, and one grade IIIb (5%) complication. The Clavian grade IIIb complication was in a patient who had a bladder neck dehiscence due to clogged SPT requiring Mitrofanoffscopy and replacement of SPT. Between 30 and 90 days postoperatively, five patients had complications, including three UTIs (Clavian grade II) and difficulty catheterizing in four patients requiring Mitrofanoffscopy (Clavian grade IIIb). One patient who was noncompliant with catheterization required percutaneous drain placement for bladder rupture (Clavian grade IIIa).

**Table 4:** Complications

Any complication	N (%)	Complication
30 day	7 (35)	
30‐90 day	6 (30)	
**30‐day complication**		
None	13 (65)	
Grade I	1 (5)	Ileus (1)
Grade II	5 (25)	UTI (2)
		Surgical site infection (1)
		Partial small bowel obstruction (1)
		Deep vein thrombosis (1)
Grade III	1 (5)	Bladder neck dehiscence (1)
**30‐90‐day complication**		
None	15 (75)	
Grade I	0 (0)	
Grade II	3 (15)	UTI (3)
Grade III	5 (25)	Bladder perforation requiring IR drainage (1)
		Difficulty catheterizing requiring mitrofanoffscopy (4)

**Figure 1:**
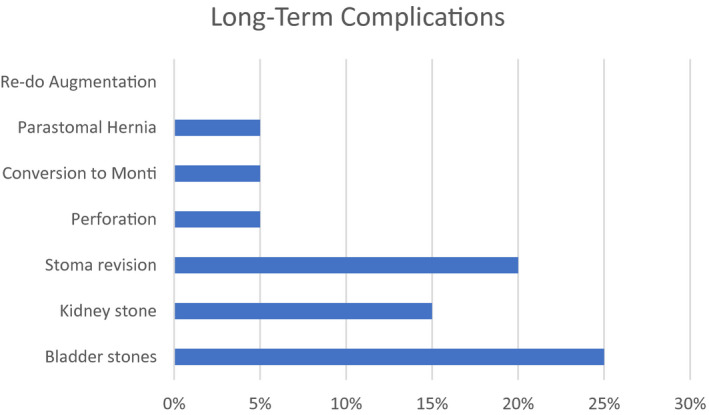
Long‐Term Complications

Figure 1 depicts the long‐term outcomes by patient during the median follow‐up of 83.1 months. Bladder stones were reported in five (25%) patients and kidney stones requiring intervention were identified in three (15%). External stoma revision at the skin level was necessary in four (20%) patients at an average time of 6 months postoperatively. Fifty‐four months after RALI, one (5%) patient required conversion to a Monti channel due to poor compliance with CIC and subsequent obliteration of APV. One patient had a parastomal hernia repair 33 months postoperatively. One of the eight (12.5%) ACE patients underwent ACE take down due to patient preference. No patients required repeat augmentation.

## 5 DISCUSSION

The robotic approach has been favored over open for decreased pain, cosmesis, and improved tissue handling without compromising outcomes across many pediatric operations.^8^, ^9^, ^10^ The laparoscopic approach to augmentation cystoplasty has not been widely accepted due to steep learning curve and increased operative time.^3^, ^11^ Robot‐assisted surgery offers the ability to shorten the learning curve and allow minimally invasive surgery to become accessible even in more complex reconstructive procedures. RALI have been shown to decrease postoperative opioid use when compared to open equivalent as well as a trend toward decreased length of stay.^2^, ^12^

Although RALIMA has its advantages, appropriate patient selection is key. Anatomic considerations, such as kyphosis leading to poor intra‐abdominal space, prior surgery leading to dense adhesions, and appendiceal anatomy can necessitate open conversion.^12^ The patient population requiring this surgery often may be wheelchair bound or have contractures, further leading to difficulty positioning. In our series we describe one patient who had a brief neuropraxia attributed to positioning. Additionally, unforeseen compliance issues regarding catheterization can lead to complications following this complex operation. Multiple complications in our series are the result of noncompliance with catheterization.

Postoperative considerations in this unique patient population regarding pain management must be considered. Many of these patients are unable to receive epidural or spinal anesthesia due to spinal dysraphism and, therefore, rely on pain control regimens which may include opioids. There are concerns that pain control regimens relying on opioids may lead to pulmonary complications as these patients often have concomitant restrictive lung disease due to kyphoscoliosis. We favor robotics for improved postoperative pain control.^13^

Concomitant procedures can be performed at time of RALI. In our series we describe eight patients who underwent ACE channel creation and six who underwent bladder neck closure or sling placement. These procedures lengthen operative time and make it difficult to assess the amount of time required to complete RALI specifically. Additional patient characteristics which lead to high variability in surgical time include patients with previous surgeries, especially patients with VP shunts, and patients with a high BMI. In our series, 40% (n = 8) had VP shunts which are associated with dense abdominal wall adhesions leading to increased operative time for adhesiolysis. The median BMI in this series was 19.9, which included three overweight patients (BMI 25‐29.9) and three patients classified as obese (BMI > 30).

RALIMA appears to be safe with comparable complication rates to the open equivalent. In this series, we report a 35% 30‐day complication rate compared to previously reported 62% for open surgery at our institution, of which 31% and 31% were grade I and III complications, respectively.^13^ Similar rates of 30‐90‐day complications were observed. Within this time period, 25% of RALI patients had complications compared to 46% open complications.^3^ Of the 25% who had complications after RALI, 15% had Clavian grade II and 25% grade III compared to open 23% clavian grade 1, 8% grade 2, and 15% grade III.

**Figure 2:**
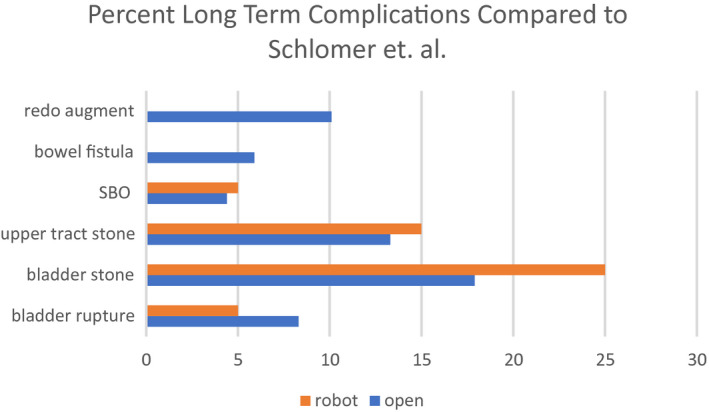
Incidence of long term complications when compared open surgery (Schlomer et al)

With a median follow‐up of 83.1 months, we note a 25% incidence of bladder stones, 15% upper tract stones, 5% incidence of bladder rupture, and 5% small bowel obstruction which is similar to previously reported data from a large database with a 10‐year follow‐up period as displayed in Figure 2.^14^ Bladder capacity was increased on average 244% in this series. RALIMA patients did not require re‐augmentation, compared to reports of 5.2‐13.4% of open operations requiring re‐augmentation.^14^

Operative times, although improving with increased experience, continue to be longer than the open alternative. The unique variability of procedures/anatomy and complexity of each patient makes it challenging to report and predict a uniform operative time. Our operative time has decreased from our first case, 623 minutes, to our most recent case, 360 minutes. Despite improvements in operative technique, the open alternative at our institution remains significantly shorter with 287 minutes of mean operative time. Contemporary open augmentation enteroscystoplasty times are not readily available in the literature. However, one group noted an average of 234 minutes for extraperitoneal approach and 336 minutes for intraperitoneal approach.^1^ Analysis of the NSQIP data by McNamara et al revealed a median operative time of 426 minutes for augmentation ileocystopasty with appendicovesicostomy, 318 minutes for an augmentation alone, and 234 minutes for appendicovesicostomy only.^15^ Although operative time remains the major criticism of the robotic approach, with increasing experience, this gap is closing.

**Figure 3:**
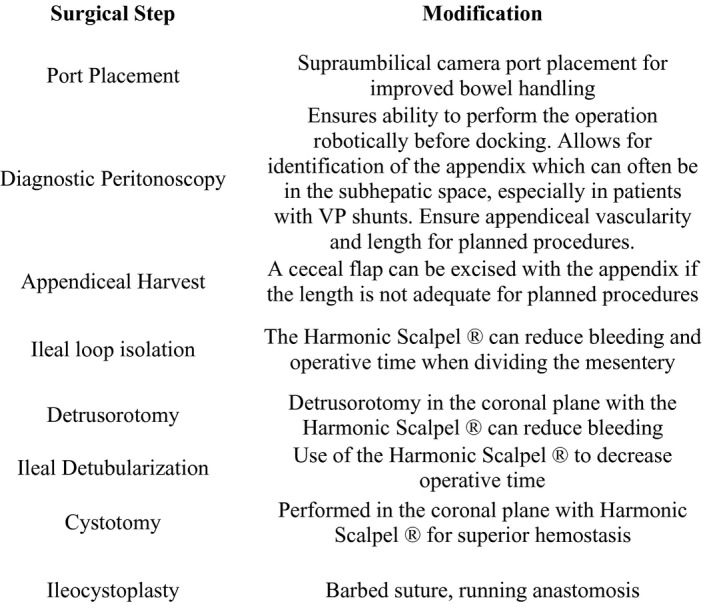
Modifications to UChicago technique

The following modifications have enabled a shorter operative time as displayed in Figure 3. The supraumbilical camera port placement allows for easier handling of the bowel and appendix. The use of the harmonic scalpel has improved operative times when performing the cystotomy, detubularizing the ileum and dividing the mesentery. We find the use of this device has superior hemostasis when compared to the monopolar/bipolar robotic energy sources. Making the detrusorotomy in the coronal plane is another way to decrease bleeding and therefore operative time. Utilizing a port site for the APV can also aid in decreasing operative time. Additionally, percutaneous bowel stay sutures and traction stitches allow for easier and faster bowel anastomosis. We prefer a running hand‐sewn anastomosis and have had good results with this technique. When performing the bowel‐bladder anastomosis, we now use a running barbed 2‐0 Quill^TM^ suture.

The RALIMA has been shown to be as safe as an open augmentation, however, it does have longer operative times. We describe 20 patients undergoing RALIMA by a single surgeon with long‐term outcomes. In our experience, operative times have been to be decreasing with increased experience and the aforementioned changes to our operative technique.
